# Laboratory confirmation of Buruli ulcer cases in Ghana, 2008-2016

**DOI:** 10.1371/journal.pntd.0006560

**Published:** 2018-06-05

**Authors:** Dorothy Yeboah-Manu, Sammy Yaw Aboagye, Prince Asare, Adwoa Asante-Poku, Kobina Ampah, Emelia Danso, Evelyn Owusu-Mireku, Zuleihatu Nakobu, Edwin Ampadu

**Affiliations:** 1 Department of Bacteriology, Noguchi Memorial Institute for Medical Research, University of Ghana, Accra, Ghana; 2 National Buruli Ulcer Control Program, Ghana Health Service, Accra, Ghana; University of Tennessee, UNITED STATES

## Abstract

**Background:**

Buruli ulcer (BU), a necrotizing skin infection caused by *Mycobacterium ulcerans* is the third most important mycobacterial disease globally after tuberculosis and leprosy in immune competent individuals. This study reports on the retrospective analyses of microbiologically confirmed Buruli ulcer (BU) cases in seventy-five health facilities in Ghana.

**Method/Principal findings:**

Pathological samples were collected from BU lesions and transported either through courier services or by car directly to the laboratory. Samples were processed and analysed by IS*2404* PCR, culture and Ziehl-Neelsen staining for detection of acid-fast bacilli. From 2008 to 2016, we analysed by PCR, 2,287 samples of 2,203 cases from seventy-five health facilities in seven regions of Ghana (Ashanti, Brong Ahafo, Central, Eastern, Greater Accra, Northern and Volta). The mean annual positivity rate was 46.2% and ranged between 14.6% and 76.2%. The yearly positivity rates from 2008 to 2016 were 52.3%, 76.2%, 56.7%, 53.8%, 41.2%, 41.5%, 22.9%, 28.5% and 14.6% respectively. Of the 1,020 confirmed cases, the ratio of female to male was 518 and 502 respectively. Patients who were 15 years of age and below accounted for 39.8% of all cases. The median age was 20 years (IQR = 10–43). Ulcerative lesions were 69.2%, nodule (9.6%), plaque (2.9%), oedema (2.5%), osteomyelitis (1.1%), ulcer/oedema (9.5%) and ulcer/plaque (5.2%). Lesions frequently occurred on the lower limbs (57%) followed by the upper limbs (38%), the neck and head (3%) and the least found on the abdomen (2%).

**Conclusions/Significance:**

Our findings show a decline in microbiological confirmed rates over the years and therefore call for intensive education on case recognition to prevent over-diagnosis as BU cases decline.

## Author summary

Buruli ulcer (BU), a necrotizing skin disease caused by *Mycobacterium ulcerans*, is currently reported in 33 countries, with the greatest disease burden mostly in West African countries along the gulf of Guinea. The lack of pain associated with BU disease enhances delay in seeking medical treatment that could result to complications. The current existing control strategy is early case detection. Previously BU diagnosis was based solely on clinical evidence by a healthcare worker, however, since other skin conditions present similar clinical signs as BU there is the need for further laboratory diagnosis. We microbiological confirmed all clinically diagnosed cases by IS*2404* PCR, and Ziehl-Neelsen. We found that over 50% of the clinically diagnosed cases were not BU, thereby averting any unnecessary antimycobacterial treatment with the associated side effects.

## Introduction

Buruli ulcer (BU), a necrotizing skin and soft tissue disease, is caused by the environmental pathogen *Mycobacterium ulcerans*. BU is the third most important mycobacterial disease after tuberculosis and leprosy in immunocompetent individuals [1]. Currently, BU has been reported in 33 countries worldwide, mainly with tropical climates, and more than two thirds of the global cases reported in West African countries along the gulf of Guinea particularly Côte d′Ivoire, Ghana, Benin and Cameroon [2]. The disease has variable clinical presentation based on geography; in the pacific regions BU may start as a papule, however, in West and Central Africa it may start as a painless nodule without the involvement of subcutaneous tissues [1]. The lack of pain that characterizes the initial stage of the disease pathogenesis, cultural practices and socio-economic factors [3, 4] results in delay in seeking medical care [5]; early clinical forms such as the nodule gradually erode leaving a well-demarcated ulcer with wide undermined edges due to the cytopathic action of the plasmid-encoded macrolide toxin, mycolactone [6, 7]. BU is classified into three categories in terms of severity: Category I, a single lesion < 5 cm in diameter, Category II, a single lesion 5–15 cm in diameter and Category III, a single lesion >15 cm in diameter, multiple lesions, critical sites, and osteomyelitis [8].

The epidemiology of BU in endemic countries is not entirely known, due to the focal distribution of cases, late reporting of cases and lack of health facilities including laboratory expertise and infrastructure for case confirmation in endemic countries of Africa. In Ghana, the first passive surveillance system reported about 1,200 BU cases between 1993 and 1998 and more than 9,000 BU cases were also reported between 2004 and 2014 [9, 10]. A nation-wide active case search that was conducted in 1999 found BU in all the 10 administrative regions of Ghana with an overall prevalence of 20.7 per 100,000 of the population [9]. Currently, BU control in Ghana is mainly through early case detection [9, 11] and clinical diagnosis at peripheral health facilities designated by the National Buruli Ulcer Control Program (NBUCP) followed by laboratory confirmation and subsequent antimycobacterial therapy.

Prior to 2004, surgical debridement of infected necrotic tissues and subsequent skin grafting to correct deformities were the main treatment options [12, 13]. The outcome of such an invasive procedure was not certain since the extent of excision was the sole prerogative of the clinician. Furthermore, lack of surgical facilities in the BU endemic areas, high cost of surgical procedures and prolonged hospitalization after surgery lasting often more than 3 months posed as a major socio-economic burden in the affected communities and discouraged a number of patients in seeking medical treatment [14]. Based on findings from a clinical trial initiated by the World Health Organization (WHO), the recommended treatment regimen is daily oral rifampicin and intramuscular injection of streptomycin for 56 days with surgery as an adjunct for improving wound healing and correction of deformities [1, 2].

The introduction of antimycobacterial therapy made laboratory confirmation of presumptive cases very critical to avoid misdiagnosis and unnecessary antibiotics administration, albeit several studies have previously reported cases that were treated on clinical grounds only but later found not to be BU but other conditions [15–17]. Nevertheless, the infrastructure and technical expertise for the gold standard method, which is polymerase chain reaction (PCR) detection of the insertion sequence IS*2404*, is nonexistent within the Ghana Health Service (GHS) facilities. Thus the GHS requested the Noguchi Memorial Institute for Medical Research (NMIMR) to assist in laboratory confirmation. Here we report on findings from a retrospective analysis of samples tested in our laboratory from 2008 to 2016.

## Methods

### Ethical issues

Samples were collected for analysis based on the national and World Health Organization guidelines for case confirmation. The procedures for sample handling and laboratory analysis was reviewed and approved by the institutional review board of the Noguchi Memorial Institute for Medical Research (NMIMR) (Federal-wide Assurance number FWA00001824). All adult participants provided informed written consent, and a parent or guardian of any child participant provided informed written consent on the child’s behalf.

### Preparation for specimen collection and transportation

**Fig 1:**
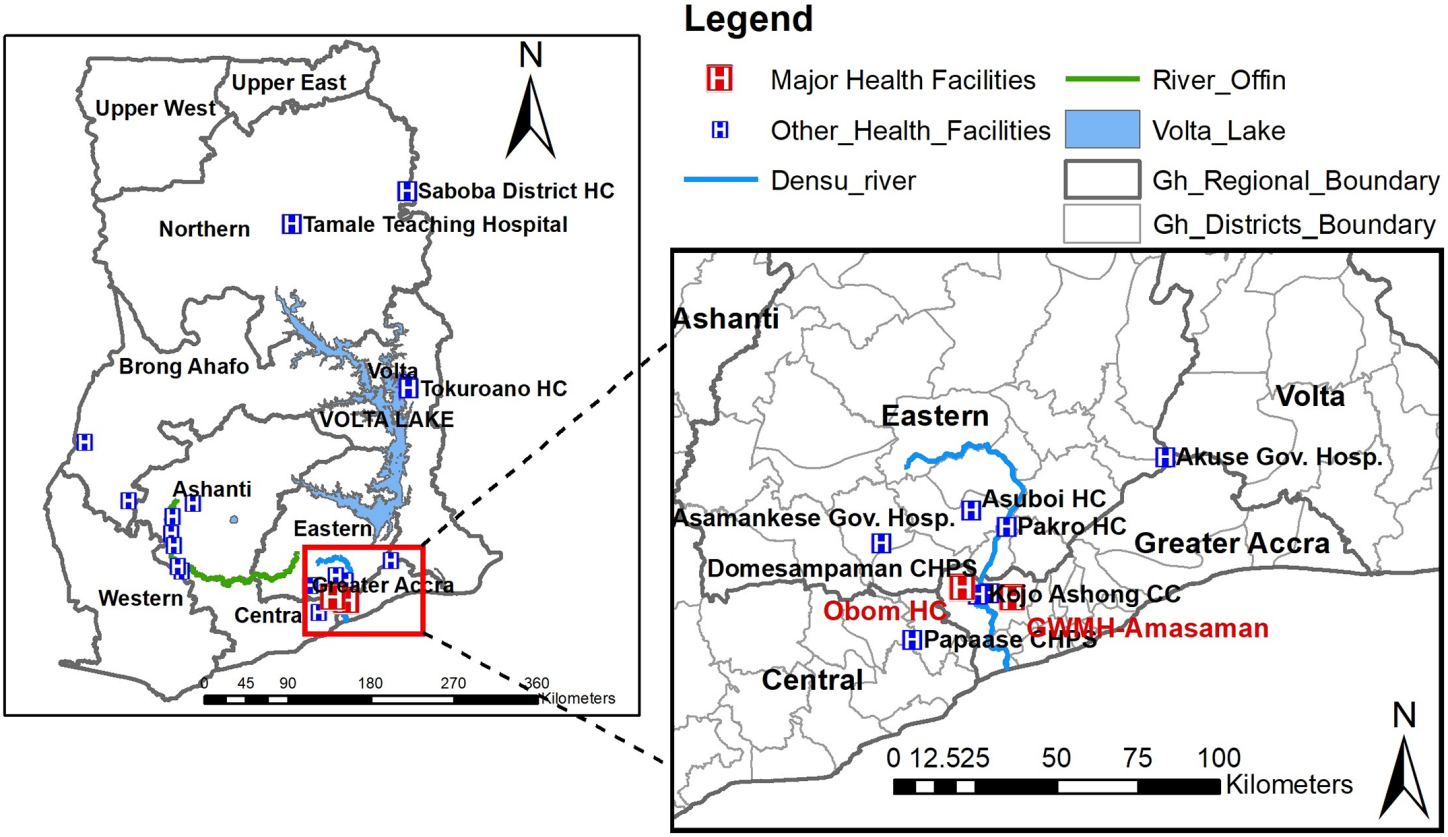
Map of Ghana showing regions of sample receipt. The background map was created using the ArcMap program in ArcGIS v.10.2 software.

The study was a retrospective one and prior to specimen collection from seventy-five selected health facilities across the country (Fig 1) designated by the National Buruli Ulcer Control Program Ghana (NBUCP). These health facilities were chosen by the NBUCP to manage BU cases across the country. The NBUCP first organized two separate workshops to build the capacity of laboratory staffs involved in the management of BU. The first workshop was conducted in 2007 in the Eastern regional capital, Koforidua and the second was conducted in the Ga West Municipal Hospital (GWMH), Amasaman of the Greater Accra region in 2008. During both workshops, health staffs comprising clinicians, nurses, laboratory staff, and diseases control officers were trained on how to appropriately collect clinical specimens from the various clinical forms of BU using swab stick and Fine Needle Aspirate (FNA) [8]. Participants were also trained on the packaging of clinical specimen and transportation under a cold chain system [8]. Also between 2009 and 2014, the Stop BU project at NMIMR as a support to the NBUCP, conducted quarterly early case search activities in these selected facilities. In addition, the Ghana Health Service also engaged courier services for the transportation of clinical specimen to the laboratory. On the average, specimens collected from within the Greater Accra region were received within 12 hours upon collection and for specimens outside Greater Accra region, the average transport time was less than 24 hours. Weekly, a team from the reference laboratory visited the two major health facilities located in the Greater Accra region (Fig 1). Samples taken prior to the team’s visit, together with newly sampled lesions were taken along for laboratory confirmation at NMIMR.

### Pathological specimens

Samples received usually for ulcerative lesions were two swab specimens from the undermined edges of the lesions while one fine needle aspirate (FNA) in 500 μl phosphate buffered saline (PBS) for pre-ulcerative lesions were also received. Punch and surgical biopsies were also received in some instances. All specimens were received in a well packaged specimen collection bags and most were together with the sample collection forms (BU 04 form and NMIMR laboratory specific form).

### Laboratory analysis

#### Sample processing

Briefly, swab specimens from the same lesion of a patient were pooled together, soaked for 30 minutes in a tube containing sterile 2ml PBS and ten 3-mm-diameter undrilled glass beads (Merck, Darmstadt, Germany). The specimen was then vortexed at full speed for 2 minutes until all the trapped specimens have been released into solution. Punch and surgical biopsies were homogenized and suspended in 2ml PBS. The suspension was then split into two portions one portion for culture and microscopy, and the other for PCR.

#### PCR

Deoxyribonucleic acid (DNA) was extracted using the Qiagen DNA mini prep kit (Qiagen, Hilden, Germany), following the manufacturer’s instruction. Amplification of a 515-bp product was performed using the primers MU1-new (5′-GAT CAA GCG TTC ACG AGT GA-3′) and MU2 (5′-GGC AGT TAC TTC ACT GCA CA-3′).^10^ The 50-μl PCR contained 5 μl 10× PCR buffer with 15 mM MgCl_2_ (QIAGEN), primers MU1 and MU2 at a final concentration of 1 μM, deoxynucleoside triphosphates (200 μM each) and 2.5 U *Taq* polymerase (QIAGEN), and 1 ng of *M*. *ulcerans* genomic DNA [18]. The amplification conditions were as previously indicated [11] and the amplicons detected after ethidium bromide staining.

#### Smear microscopy and culture

The sample suspension was decontaminated by adding equal volume of 5% oxalic acid and incubated at room temperature for 30 minutes with occasional vortexing [19]. The reaction was stopped by neutralizing with excess sterile PBS, after which the mixture was centrifuged for 30 minutes at 3000 g. The supernatant was decanted; the pellet re-suspended in 300 ul of sterile PBS and 100 ul of specimen suspensions was inoculated in duplicate on Löwenstein-Jensen (LJ) medium slants supplemented with polymixin B, amphotericin B, nalidixic acid, trimethoprim, and azlocillin (PANTA) and mycobactin J [20]. The inoculated culture tubes were incubated at 32°C and observed for macroscopic growth for 6 months, at which time they were discarded. Culture tubes were read daily during the first week, for contamination, and thereafter were read weekly [20]. Suspected *M*. *ulcerans* were confirmed as described previously [21]. The remaining suspension was used to prepare a smear for acid fast bacilli (AFB) detection using the Ziehl Neelsen (ZN) procedure. The slides were graded according to the International Union against Tuberculosis and Lung Diseases standard [22].

### Identification of mycobacterial isolates

The isolates confirmed as AFB positive were harvested, killed by heating at 95°C for 30 min, and used for genomic DNA extraction as previously described [23]. A 441-bp portion of mycobacterial heat shock protein 65 (hsp65) was amplified using the *Mycobacterium* genus-specific TB-11 5’-ACC AAC GAT GGT GTG TCC AT-3’ and TB-12 5’-CTT GTC GAA CCG CAT ACC CT- primers [24], as described previously [25]. The PCR mixture contained 5μl of a 1:100 dilution of template DNA, 0.25μM concentrations of each primer, 6 μl of Q-solution, 3μl of 10X buffer, 200 μM (each) dATP, dCTP, dGTP, and dTTP (Pharmacia Biotech), 1.5Mm MgCl2, and 0.5 U of Fire *Taq* polymerase, in a total volume of 100 μl. Amplification was performed using 32 cycles of 5 min at 94°C, 30 s at 94°C, 30 s at 60°C, 1 min at 72°C, and 10 min at 72°C, in an Applied Biosystems 2720 thermal cycler [18]. Amplified products were confirmed by gel electrophoresis. The amplified PCR product (40 μl) was sequenced by outsourcing. The generated sequences were edited using Codon Code Aligner 6.0.2 software to remove vector sequences, and species were identified by using NCBI Microbial Nucleotide BLAST, using default settings [26].

### Data analyses

Clinical and demographic data were retrieved from all study participants using the specimen collection form and entered into Microsoft Access with validation to correct for entry errors. All statistical analyses were carried out using the Stata statistical package version 14.2 (Stata Corp., College Station, TX, USA). The chi square test for trend (ptrend) was explored to assess the significance of the observed decreasing proportion of laboratory cases over the years. A P-value < 0.05 was considered significant.

## Results

### Clinical specimens for BU diagnosis

A total of 2,287 clinical specimens from 2,203 BU presumptive cases were received from 75 health facilities from seven out of the ten regions of Ghana (Fig 1). Pathological specimens received were 1,892 (82.7%) swabs, 384 FNA (16.8%) and 11 (0.5%) biopsies (Tables 1 and 2). The specimens were from 1,637 (74.2%) ulcerative lesions, 250 (8.4%) nodules, 65 (2.9%) plaque, 42 (1.9%) oedema and 26 (1.2%) with osteomyelitis (Table 1). Samples collected from multiple clinical forms include: ulcer and oedema 84 (3.8%), ulcer and plaque 77 (3.5%) and ulcer and osteomyelitis 22 (0.9%) as indicated in Table 1. The numbers of males were 1117 (50.7%) and females 1086 (49.3%). The median age was 22 years (range: 0.3–100 years).

**Table 1:** Clinical characteristics of BU cases confirmed by IS*2404* PCR.

Characteristics	No. Cases	Confirmed N (%)	Cat I N (%)	Cat II N (%)	Cat III N (%)	Not stated N (%)
**Sex**						
Male	1117	502 (49.2)	137 (27.3)	74 (14.7)	172 (34.3)	119 (23.7)
Female	1086	518 (50.8)	133 (25.7)	84 (16.2)	185 (35.7)	116 (22.4)
**Age**						
0–15*	880	406 (46.1)	141 (34.7)	70 (17.2)	113 (27.8)	82 (20.2)
16–30	398	188 (47.2)	45 (23.9)	34 (18.1)	73 (38.8)	36 (19.2)
31–45	342	158 (46.2)	32 (20.3)	29 (18.4)	63 (39.8)	34 (21.5)
46–60	246	115 (46.7)	27 (23.5)	10 (8.7)	53 (46.1)	25 (21.7)
61 +	239	108 (45.2)	20 (18.5)	14 (12.9)	45 (41.7)	29 (26.9)
Not stated	98	45 (45.9)	5 (11.1)	1 (2.2)	7 (15.6)	32 (71.1)
**Lesion**						
Ulcer	1637	744 (45.4)	182 (24.5)	111 (14.9)	251 (33.7)	200 (26.8)
Nodule	250	96 (38.4)	62 (64.5)	9 (9.4)	9 (9.4)	16 (16.7)
Plaque	65	30 (46.2)	11 (36.7)	9 (30.0)	7 (23.3)	3 (10.0)
Edema	42	22 (52.4)	5 (22.7)	2 (18.2)	13 (59.1)	2 (18.2)
Osteomyelitis	26	11(42.3)	1(9.1)	4 (36.4)	5 (45.5)	1(9.1)
Ulcer/Edema	84	63(72.4)	20 (31.7)	14 (22.2)	28 (44.4)	1(1.6)
Ulcer/Plaque	77	43(55.8)	11(25.6)	9 (20.9)	19 (44.2)	4 (9.3)
Ulcer/osteomyelitis	22	11(50.0)	1(9.1)	2 (18.2)	7 (63.6)	1(9.1)
**Sample type**						
Swab	1892	852 (83.5)	192 (22.5)	132 (15.5)	319 (37.4)	209 (24.5)
FNA	384	160 (15.7)	78 (48.7)	26 (16.3)	29 (18.1)	27 (16.9)
Biopsy	11	8 (0.8)	0 (0)	0 (0)	6 (75.0)	2 (25.0)

* Significant variation among ≤15 year group and any other age groups confirmed for BU (p<0.001)

**Table 2:** BU cases diagnosed by clinical specimen.

	Swab		FNA		Biopsy	
Year	ZN [N (%)]	PCR [N (%)]	ZN [N (%)]	PCR [N (%)]	ZN [N (%)]	PCR [N (%)]
**2008**	6/35 (17.1)	19/35 (54.3)	1/7 (14.3)	3/7 (42.8)	-	-
**2009**	26/53 (49.1)	40/53 (93.0)	2/7 (28.6)	5/7 (71.4)	2/3 (66.7)	3/3 (100)
**2010**	165/419 (39.4)	248/419 (59.2)	15/74 (20.3)	32/74 (43.2)	-	-
**2011**	121/484 (25.0)	250/484 (51.7)	6/124 (4.8)	76/124 (61.3)	0/1 (0.0)	1/1 (100)
**2012**	48/272 (17.6)	119/272 (43.8)	11/58 (18.9)	17/58 (29.3)		
**2013**	34/232 (14.6)	98/232 (36.0)	8/44 (18.2)	16/44 (36.4)	1/6 (16.7)	3/6 (50.0%)
**2014**	24/209 (11.5)	48/209 (22.9)	3/43 (6.9)	9/43 (20.9)	1/1 (100)	1/1 (100)
**2015**	14/149 (9.4)	22/149 (14.7)	0/21(0.0)	2/21 (9.5)	-	-
**2016**	3/42 (7.1)	7/42 (16.6)	0/6 (0.0)	0/6 (0.0)	-	-
**Total**	**441/1895 (23.3)**	**852/1895 (44.9)**	**46/384 (11.9)**	**160/384 (41.7)**	**4/11 (36.4)**	**8/11 (72.7)**

### Cases confirmed by IS*2404* PCR and smear microscopy

The summary results for samples analysed by both PCR and ZN are presented in Tables 3, 4 and 5. Of the total 2,203 BU cases analysed, 1020 (46.3%) were positive for IS*2404* PCR and 491 (22.3%) were positive for smear microscopy by ZN staining. Out of the 1020 IS*2404* PCR positives, 852 (38.7%) were swabs, 160 (7.3%) FNA and 8 (0.8%) were biopsies. The 491 ZN positive cases comprised of 441 (89.9%) swabs, 46 (9.4%) FNA and 4 (0.8%) biopsies. As indicated in Table 3, out of the 1020 confirmed by IS*2404* PCR, AFB were detected among 344 (33.7%) of these PCR positives. Further analyses showed that 645 (63.2%) samples that were negative by ZN staining were positive by PCR (Table 3).

**Table 3:** BU case confirmation by PCR and ZN.

Year	No. of Cases	PCR Positive	ZN Positive	Both PCR and ZN Positive	ZN Negative but PCR Positive
2008	42	22	7	1	2
2009	63	48	30	25	18
2010	495	281	180	99	175
2011	609	327	127	96	230
2012	330	136	59	44	86
2013	283	117	43	37	79
2014	249	58	28	25	33
2015	84	24	14	14	18
2016	48	7	3	3	4
**Total**	**2203**	**1020**	**491**	**344**	**645**

**Table 4:** Regional BU cases confirmed by IS*2404* PCR.

Yearly cases N (% Confirmed by IS*2404* PCR)										
Region	2008	2009	2010	2011	2012	2013	2014	2015	2016	Total (%)
Ashanti	0	0	2/5 (40)	36/65 (55.4)	0	26/49 (53.1)	8/37 (21.6)	2/5 (40.0)	0/1 (0)	74/162 (45.7)
Brong Ahafo	0	0	48/57 (84.2)	18/52 (34.6)	0	0	1/1 (100)	0	0	67/110 (60.9)
Central	0	0	6/7 (85.7)	7/14 (50)	3/9 (33.3)	6/17 (35.3)	3/15 (20)	2/19 (10.5)	0/3 (0)	27/84 (32.1)
Eastern	0	7/11 (63.6)	50/100 (50)	53/111 (47.7)	34/81 (42.0)	10/20 (50.0)	4/18 (22.2)	1/8 (12.5)	4/21 (19.0)	163/375 (43.5)
Greater Accra	22/42 (52.4)	41/52 (78.8)	173/323 (53.5)	202/350 (57.7)	97/232 (41.8)	71/184 (38.6)	41/176 (23.3)	18/49 (36.7)	3/23 (13.0)	668/1511 (44.2)
Northern	0	0	2/3 (66.7)	0	0	2/2 (100)	0	0	0	4/5 (80.0)
Volta	0	0	0	11/17 (64.7)	2/8 (25.0)	2/10 (20.0)	1/2 (50.0)	1/3 (33.3)	0	17/40 (42.5)
**Total**	**22/42**	**48/63**	**281/495**	**327/609**	**136/330**	**117/283**	**58/249**	**24/84**	**7/48**	**1020/2203 (46.3)**

**Table 5:** Regional BU cases diagnosed by ZN.

Yearly cases N (% Diagnosed by Smear Microscopy)										
Region	2008	2009	2010	2011	2012	2013	2014	2015	2016	Total (%)
Ashanti	0	0	3/5 (60)	8/65 (12.3)	0	8/49 (16.3)	6/37 (16.2)	0/5 (0)	0/1 (0)	25/162 (15.4)
Brong Ahafo	0	0	25/57 (43.8)	7/52 (13.5)	0	0	0/1 (0)	0	0	32/110 (29.1)
Central	0	0	1/7 (14.3)	1/14 (7.1)	0/9 (0)	3/17 (17.6)	0/15 (0)	0/19 (0)	0/3 (0)	5/84 (5.9)
Eastern	0	6/11 (37.5)	29/100 (29.0)	23/111 (20.7)	15/81 (18.5)	1/20 (5.0)	2/18 (11.1)	0/8 (0)	2/21 (9.5)	78/375 (20.8)
Greater Accra	7/42 (16.7)	24/52 (45.3)	122/323 (37.7)	85/350 (24.3)	43/232 (18.6)	30/184 (16.2)	20/176 (11.4)	13/49 (26.5)	1/23 (3.3)	345/1511 (22.8)
Northern	0	0	0/3 (0)	0	0	0/2 (0)	0	0	0	0/5 (0.0)
Volta	0	0	0	3/17 (17.6)	1/8 (12.5)	1/10 (10.0)	0/2 (0)	1/3 (33.3)	0	6/40 (15.0)
**Total**	**7/42 (52.3)**	**30/63**	**180/495**	**127/609**	**59/330**	**43/283**	**28/249**	**14/84**	**3/48**	**491/2203 (22.3)**

**Fig 2:**
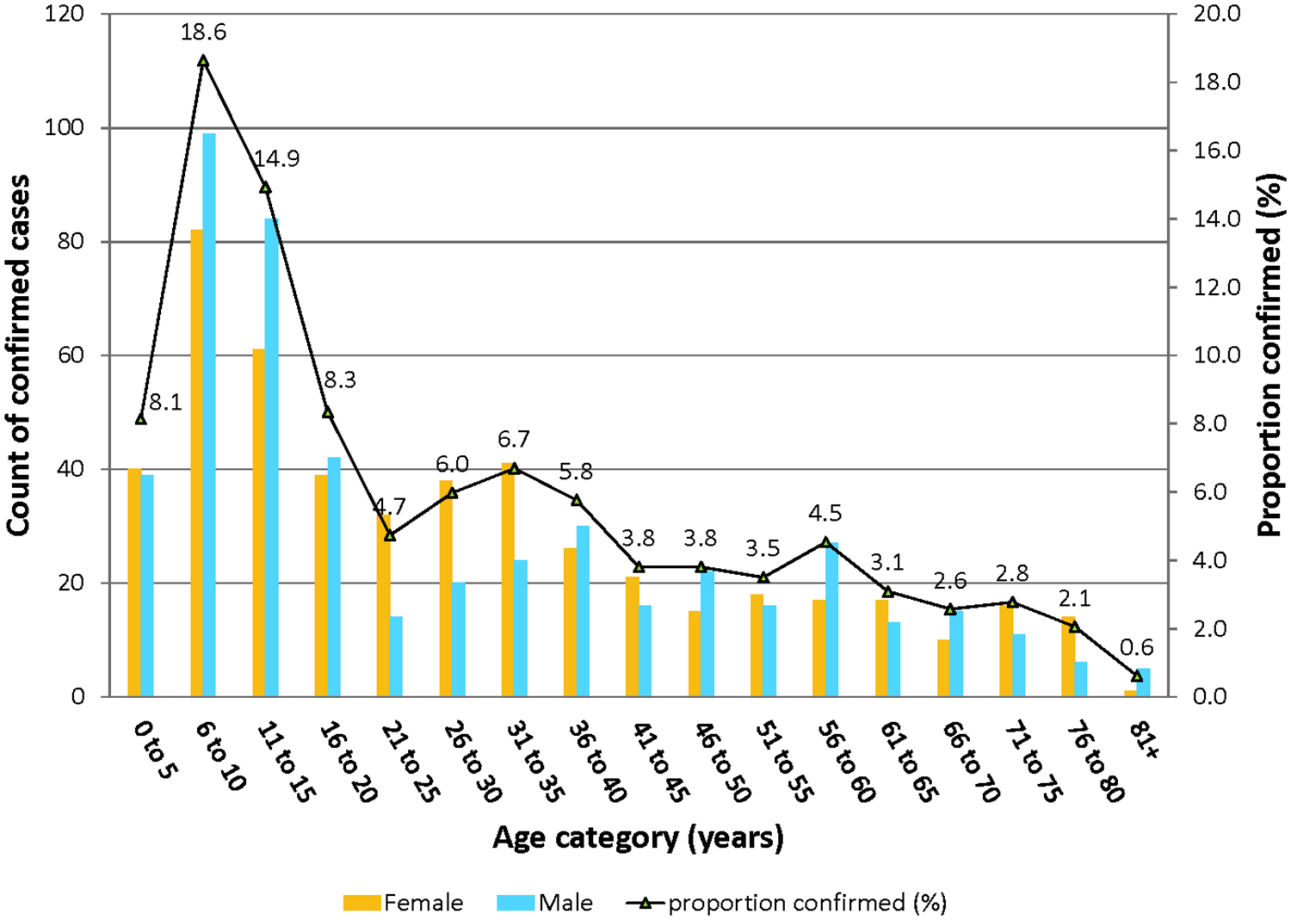
Age and gender distribution of confirmed BU cases in Ghana.

Among IS*2404* PCR confirmed cases, 502 (49.2%) were males and 518 (50.8%) were females (Table 1). The age range was 1–100 years with median age 20 years. In 45 (4.4%) of the confirmed cases, age records were not available on sample collection forms. Children below 15 years of age formed 39.8% of all cases confirmed. Comparing this lower age category (≤15 years), confirmed BU cases in each other age category was significantly lower (p<0.001) (Fig 2).

**Fig 3:**
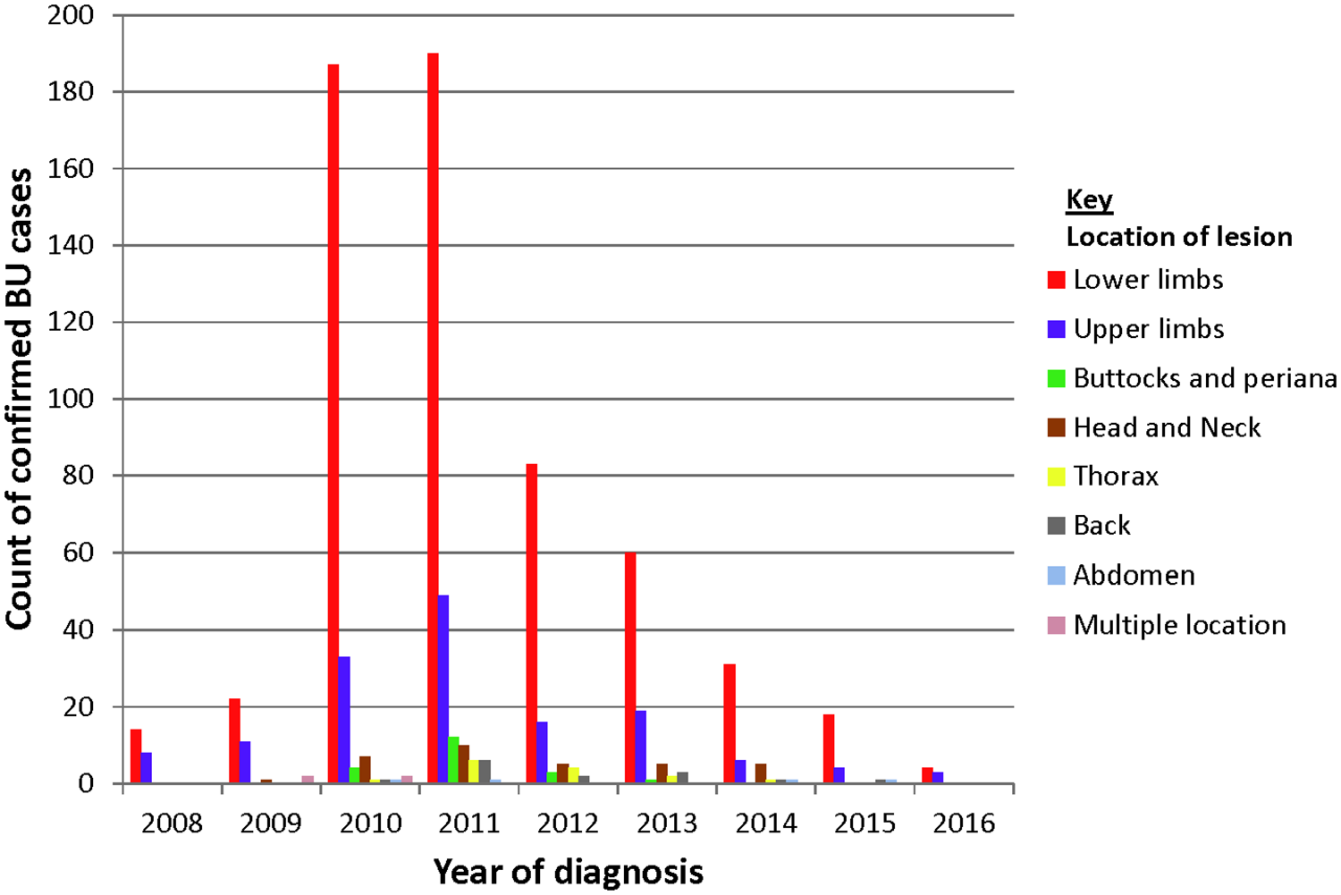
Lesion sites of confirmed BU cases.

Of the 1,020 confirmed cases, 744 (72.9%) were ulcerative lesions, 96 (9.4%) nodules, 30 (2.9%) plaque, 22 (2.2%) edema and 11 (1.1%) were osteomyelitis. Some confirmed cases presented lesions with multiple clinical forms including ulcer and edema 63 (6.2%), ulcer and plaque 43 (4.2%) and ulcer and osteomyelitis 11 (1.1%). The lesions categories presented were: 270 (26.5%) category I, 158 (15.5%) category II and 357 (35.0%) category III while 235 (23.0%) had no information on lesion category. Although BU lesions were broadly distributed in all body parts, BU lesion were mostly located on lower limbs 609/1020 (59.7%) followed by upper limbs 160/1020 (15.6%) (Fig 3). Lesion located on lower limbs was consistently highest; 2008 [14/22 (63.6%)], 2009 [22/48 (45.8%)], 2010 [187/281 (66.5%), 2011 [190/327 (58.1%)], 2012 [83/136 (61.0%)], 2013 [60/117 (51.3%)], 2014 [31/58 (53.4%)], 2015 [18/24 (75.0%)] and 2016 [4/7 (57.1%)]. (Fig 3)

### Geographical distribution of confirmed BU cases

**Fig 4:**
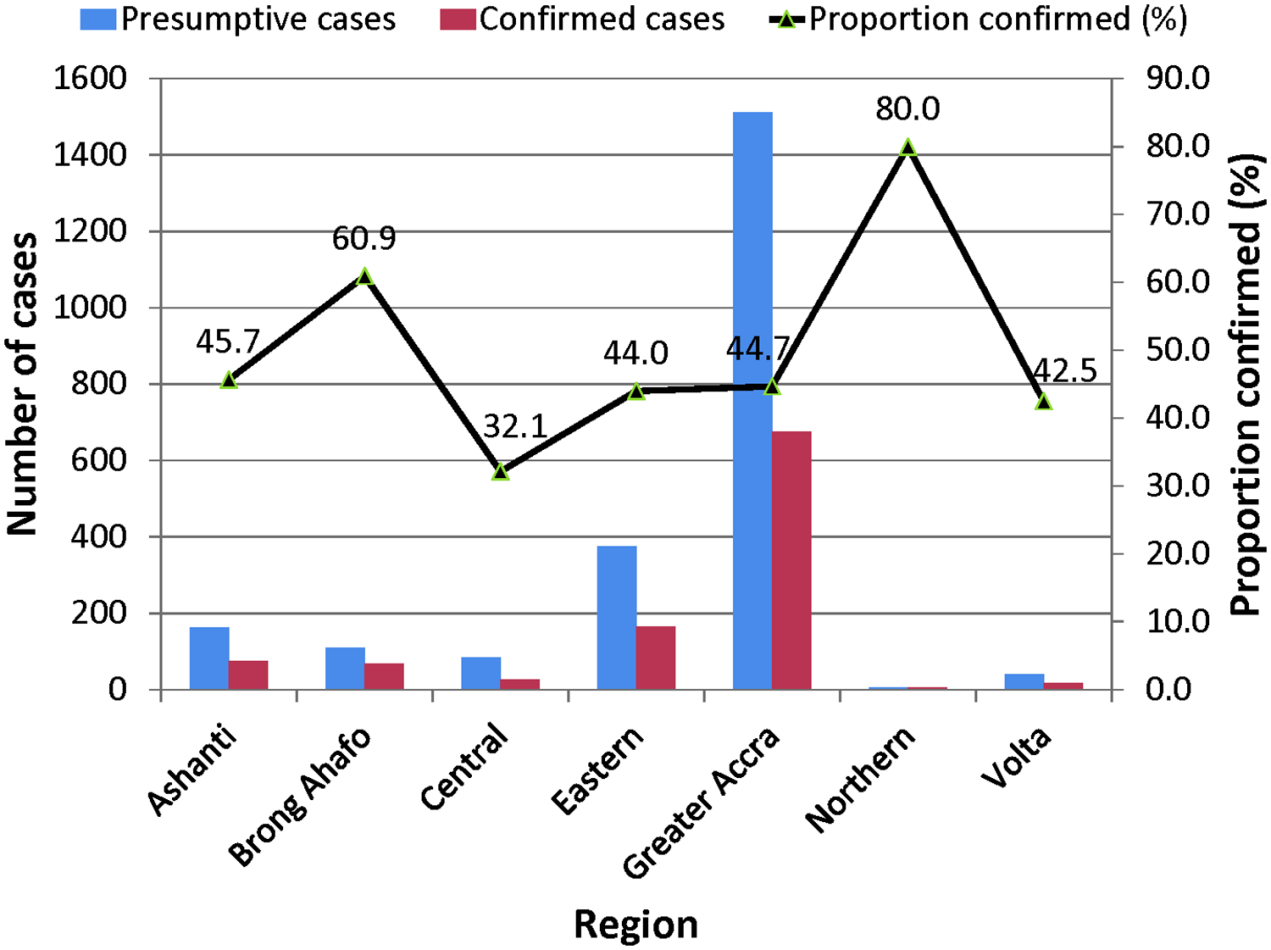
BU case confirmation rate by region.

**Fig 5:**
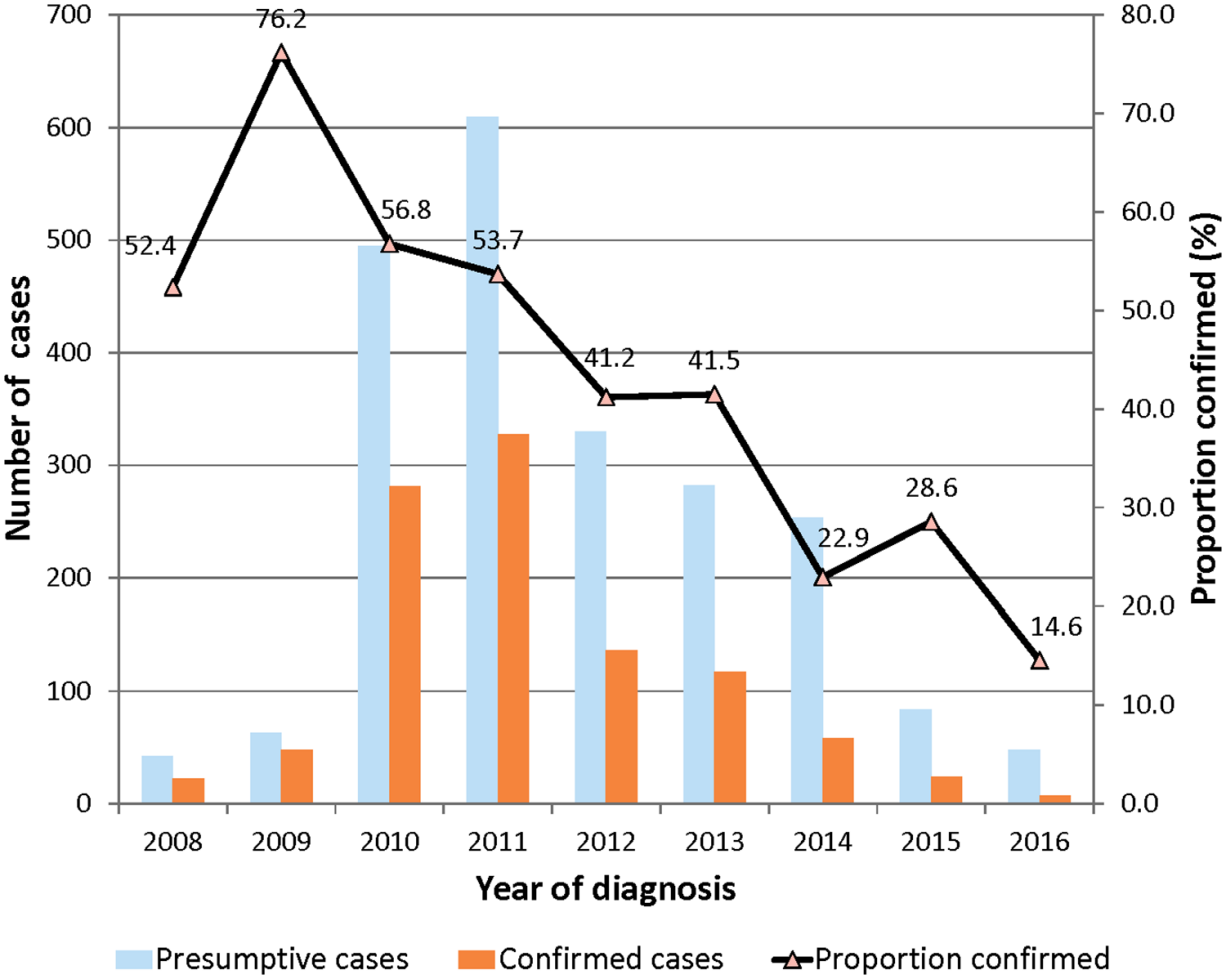
Annual BU case confirmation rate.

Altogether, case samples were received from seven regions in Ghana and were confirmed as BU by PCR. Among the seven regions of Ghana, cases distribution are as follows, Ashanti 74, Brong Ahafo 67, Central 27, Eastern 163, Greater Accra 668, Northern 4 and Volta 17 as shown in Table 4. The regional BU confirmation rates by PCR from our data set were Ashanti 45.7%, Brong Ahafo 60.9%, Central 32.1%, Eastern 44.0%, Greater Accra 44.7%, Northern 80.0% and Volta 42.5% as indicated in (Table 4 and Fig 4). Analyses of BU cases by ZN staining method followed a similar pattern as PCR results (Table 5). Overall, a decreasing trend of proportion of confirmed BU cases (p ≤ 0.001) was reported over the study period from (76.8%) by the end of 2009 reaching 7/48 (14.6%) by the end of 2016 as shown in Table 6 and Fig 5.

**Table 6:** Yearly BU case confirmation by PCR.

Variables	2008	2009	2010	2011	2012	2013	2014	2015	2016	Total
No of sampled patients	42	63	495	609	330	283	249	84	48	2203
No of patients positive for PCR	22	48	281	327	136	117	58	24	7	1020
Yearly positive rate	52.3	76.2	56.8	53.7	41.2	41.3	23.3	28.6	14.6	46.3

### Mycobacterial identity by *Hsp65* sequencing

Of the 1020 IS*2404* PCR positive specimens cultured, macroscopic growth was detected for only 316 (30.9%) after 6 months of incubation. Although ZN staining confirmed all the 316 isolates as AFB, subsequent confirmation by *hsp65* sequencing identified 244 (77.2%) as *Mycobacterium ulcerans* whilst the remaining were members of nontuberculous mycobacteria; *Mycobacterium avium* 31(43.1%), *Mycobacterium fortuitum* 28 (38.9%), and were *Mycobacterium abscessus* 13 (18.1%).

## Discussion

We confirmed only 46% of the total 2,203 BU cases by IS*2404* PCR. Our findings suggest that over 50% of the clinically diagnosed cases may not be BU. This finding calls for the need to confirm cases before they are put on antimicrobial treatment to avert putting individuals on needless antimicrobials.

Recently, we followed up on 77 cases that were historically negative for IS*2404* PCR. We observed that 86.8% of these cases wounds were completely healed and 13.2% were partially healed without any antimycobacterial treatment [27]. Similarly, there have been reports of lesions clinically diagnosed as BU but were later confirmed as tropical phagedenic ulcer, deep fungal infection, cellulitis and diabetic ulcer [15–17, 28]. It has previously been thought that diagnosis of ulcerative lesions is very straightforward due to readily recognized indolent, undermined edges lesions. However within our analyzed samples the confirmation rates of ulcerative lesions were equally low compared to other presentations of BU. Ghana introduced the current antibiotic treatment in 2006, and the policy is that all clinically recognized cases are put of SR8 without laboratory confirmation results. This practice/policy needs to be revised considering the number of reports on misdiagnosis of BU. Moreover the main bactericidal drug rifampicin (RIF) is also one of the main anti-mycobacterial agents for the treatment of tuberculosis (TB). Ghana currently has been recognized as one of the 30 most burdened TB nation due to the high HIV-TB. At the same time due to the emergence of drug resistance TB strains the use of RIF must be reduced only to needed patients. In addition, RIF is hepatotoxic [29, 30] while streptomycin [31–33] is autotoxic especially to children. Considering that a significant proportion of those affected were children below the age 16 years, we propose 1) training of clinicians involved in BU diagnosis and treatment at all levels 2) that the GHS and other health services in endemic African countries revise the initial policy of antimycobacterial treatment based on clinical diagnosis alone. As an alternative, wound care practices could be employed for all clinician-diagnosed cases as an interim arrangement till microbiological confirmation is done. We also observed that nearly 40% of the PCR-positive cases were also positive by smear microscopy after ZN staining. Currently, smear microscopy has not been included BU case management in Ghana although it is employed in routine diagnosis of TB in most peripheral laboratories in the country. We are of the opinion that smear microscopy by ZN staining has to be included as a first line diagnostic tool for BU. Of the 1020 PCR positive samples that we cultured, macroscopic growth was obtained for only 30%. Sequencing analysis confirmed 77% as *M*. *ulcerans* whilst the remaining 23% were members of the nontuberculous mycobacteria. Although culture is the full proof of viable mycobacteria the cultivation challenges such as the slow growth nature which may take about 8 to12 weeks or more, the presence of fast growing microorganism that may take over cultivation media makes culture not suitable for BU diagnosis as patients would have to wait for longer period before undergoing treatment [34].

One of the theories proposed to explain the mechanism of *M*. *ulcerans* transmission is direct contact of an exposed skin with a contaminated environmental source such as sharp leaves or through pre-existing wounds [35]. Two separate studies have shown that BU lesions mostly occur where the bones are close to the skin (shins, knees, elbows and forearms) [21, 36]. We observed a distribution pattern that supports the direct contact with an exposed skin hypothesis as 74.4% of BU lesions were restricted to the limbs; lower limb (59.7%) and upper limb (14.7%) corroborating with several other reported studies [37–42]. In the African BU endemic regions, the hot weather conditions may be a major contributory factor for this localized lesion restriction as farmers in particular are less likely to wear protective clothes during activities to enhance efficient work output; a behavior that is likely to enhance the exposure of the skin to *M*. *ulcerans* in the environment [42].

The annual BU case confirmation rates over the years indicate that BU cases are on the decline, for example, the rates increased from 52.4% in 2008 to 76.2% in 2009. The improved confirmation rates observed during this period may be attributed to the prior training activities conducted by the NBUCP to healthcare givers within the Ghana Health Service facilities on case detection and proper specimen collection. By the end of 2016, BU rates had gradually declined to 14.6%. This downward trend may be a reflection of actual reduction in BU cases or replacement of previously trained personnel with new health staff with very little or no experience in BU diagnosis. It must be emphasized that the Stop BU project at NMIMR conducted quarterly early case search activities between 2009 and 2014 when outreach activities by the NBUCP ceased. This probably might have accounted for the improved BU rates seen within those periods. In addition, due to the focal distribution nature of BU, even in endemic countries such as Ghana not all clinicians are familiar with BU. This requires routine training of clinical staff in endemic countries.

We found children 15 years and below to be a major risk group and this is in agreement with findings from other countries [43, 44] although a study in Benin showed that adults between 75 and 79 years are at high risk of developing BU [45]. We observed that the number of BU cases was low in children <5 years, which agrees with our recent studies in both Ghana and Cameroun that indicated less exposure of children in this age bracket to *M*. *ulcerans*. BU is known to affect both sexes, however; we observed that males below age ≤ 15 years were more affected than females (p-0.011). The observed differences may be due to the different recreational activities engaged in by both sexes. For instance, in African endemic countries, young males are more likely to play football shirtless and move to riverside to swim thereby having more environmental contact [42].

The limitation of the study was that all case confirmation was by gel-based PCR although many reference laboratories have switched to real-time PCR and semi-automated platforms which have a lower propensity for amplicon contamination (false-positives). However, at NMIMR, we have had no experience of contamination in our laboratory as we employed a four chamber system in the analyses of our samples. Different rooms with separate biosafety cabinets are used for sample preparation and DNA extraction whilst mastermix preparation, temperate addition and finally PCR are performed also in separate rooms.

In conclusion, laboratory confirmation of presumptive BU cases still remains an essential aspect in the management of Buruli ulcer in Ghana and needs to be included in the case management of BU as done for tuberculosis to avert misdiagnosis and unnecessary antibiotic treatment. While we agree that case confirmation by PCR may be a challenge for all cases, a simple differential diagnosis chart plus microscopy could also be beneficial for case recognition in remote centres.

## Supporting information

S1 Checklist STROBE checklist. (DOC)

S1 Form BU 04 form. (PDF)

S2 Form NMIMR laboratory specific form. (PDF)
