# Supplementary material for: Laboratory confirmation of Buruli ulcer cases in Ghana, 2008-2016
Source: PLoS Negl Trop Dis. 2018 Jun 5;12(6):e0006560. doi: 10.1371/journal.pntd.0006560 (PMC6003692; doi:10.1371/journal.pntd.0006560)
Supplement: S1 Form — (PDF) [file pntd.0006560.s002.pdf]

LABORATORY EXAMINATION REQUEST FORM-BURULI ULCER BU 04

Health Facility: ____

Name of patient:____Age__________Sex: ☐M ☐F

Address____Patient ID No:______________

Classification: **Category of lesion(s):**

☐ **New** ☐ **Category I**: A single lesion < 5 cm in diameter **Category II**: A single lesion 5 – 15 cm in diameter

☐ **Recruitment** ☐ **Category III**: A single lesion >15 cm in diameter, multiple lesions, critical sites, osteomylitis

Clinical forms(s): ☐ Nodule (N) ☐ Plaque (Q) ☐ Oedematous (E) ☐ Ulcer (U) ☐ Osteomyelitis (O) ☐ Papule (P)

Type of specimen: ☐ Swab ☐ Fine Needle Aspiration (FNA) ☐ Biopsy Date of specimen collection:

**Type of lab examination(s)**	☐ ZN	☐ PCR	☐ Culture	☐ Histopathology

Reasons for requesting lab examination

☐ New case ☐ Follow-up of a patient during antibiotic treatment (weeks of antibiotic treatment):_________

☐ Recurrent case (end of last antibiotic treatment (date/months:____________) ☐ Post treatment follow-up

________________________________ ________________________________

Name of person requesting examination Signature

Results (to be filled by laboratory) Lab Specimen No____________________

Date	**ZN**	**PCR**	**Culture**	**Histopathology**
__/__/___				
__/__/___				

Comments:____

________________________________ ________________________________ ____ / ____ / ____

Name of person providing result Name of facility Date

National Buruli Ulcer Control Programme, Ghana Health Service, P. O. Box KB 493, Accra. Tel: 021 – 686337, Fax: 021 – 686336, Email: ghanbu@4u.com.gh

printed by ACTS COMMERCIALS LTD. Te.: 021 – 300940 / 312620
