# Supplementary material for: Laboratory confirmation of Buruli ulcer cases in Ghana, 2008-2016
Source: PLoS Negl Trop Dis. 2018 Jun 5;12(6):e0006560. doi: 10.1371/journal.pntd.0006560 (PMC6003692; doi:10.1371/journal.pntd.0006560)

Specimen Collection Form, STOP Buruli Project

A. Institutional information

Health facility: ________________________________

District________________________________

B. Patient Information

Name of patient: ____________________________ Age (yrs):_____ Sex: ☐M ☐F

Health facility ID: ____________ Date of Admission: ___/___/___

Residential address/Village:

Sub-district: District:

Occupation:

Source of drinking water: Tap☐ Bore hole/well ☐ River/stream ☐ Pond ☐

Classification: ☐ New ☐Recurrent

Duration of lesion before seeking care:

History of cases in family/relatives/friends: Yes☐ No ☐ Specify ....

History of trauma at site of lesion: Yes☐ No ☐

Use of traditional treatment: Yes☐ No ☐

BCG vaccination/scar: Yes☐ No ☐

Pain: Yes☐ No☐

C. Location of lesion:

Upper limb: right☐ left☐ Abdomen☐ Back☐ Head & neck☐

Lower limb: right☐ left☐ Thorax☐ Bottucks/ perineals☐

D. Clinical form(s):

☐Nodule(N) ☐ Plaque(Q) ☐ Oedema(E) ☐Ulcer(U) ☐Osteomyelitis(O)

Category:

Date of specimen collection:___/___/___ Type of specimen: ☐Swab ☐FNA

☐Punch biopsy

☐Biopsy ☐ Blood

E. Reasons for laboratory request:

☐New patient ☐Recurrent case(last SR treatment: date /month___/____)

☐Follow up during treatment:_____ days of SR treatment

☐Follow-up after SR treatment: _______ weeks of treatment

[redacted] ☐ Histopathology ☐ Culture

_____/_____/_____

____

Date specimen sent to lab **Name of person making request**

II. This section should be completed by the laboratory

Date specimen received in laboratory ___/___/____

Summary Results

**Examination**	[redacted]	[redacted]	Date
ZN	☐	☐	__/__/__
PCR	☐	☐	__/__/__
Histopathology	☐	☐	__/__/__
Culture	☐	☐	__/__/__

__________________________________ ____/____/_____

____

Name of person providing results **Date**
Signature

NMIMR ID #

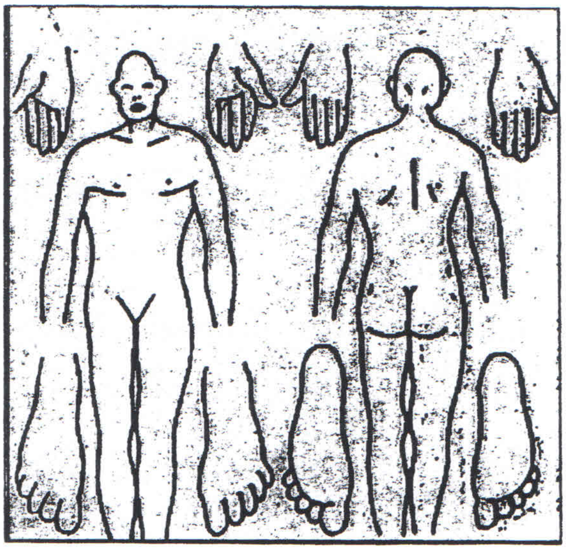

Supplement: S2 Form — (PDF) [file pntd.0006560.s003.pdf]
